# Thromboxane A_2_ Modulates de novo Synthesis of Adrenal Corticosterone in Mice via p38/14‐3‐3γ/StAR Signaling

**DOI:** 10.1002/advs.202307926

**Published:** 2024-03-09

**Authors:** Shuai Yan, Yuanyang Wang, Bei Wang, Shengkai Zuo, Ying Yu

**Affiliations:** ^1^ Department of Pharmacology Tianjin Key Laboratory of Inflammatory Biology State Key Laboratory of Experimental Hematology Key Laboratory of Immune Microenvironment and Disease (Ministry of Education) The Province and Ministry Co‐sponsored Collaborative Innovation Center for Medical Epigenetics School of Basic Medical Sciences Tianjin Medical University Tianjin 300070 P. R. China; ^2^ Division of Endocrinology, Diabetes, and Metabolism Beth Israel Deaconess Medical Center Harvard Medical School 330 Brookline Avenue Boston Massachusetts 02115 USA; ^3^ Department of Biopharmaceutics Tianjin Key Laboratory of Technologies Enabling Development of Clinical Therapeutics and Diagnostics School of Pharmacy Tianjin Medical University Tianjin 300070 P. R. China

**Keywords:** 14‐3‐3γ, adrenal corticosterone, p38, steroidogenic acute regulatory protein, thromboxane receptor

## Abstract

Prostanoids are endogenous lipid bioactive mediators that play essential roles in physiological processes such as glucocorticoid secretion. Here, it is found that the thromboxane (Tx)A_2_ receptor (TP) is highly expressed in the adrenal cortex of mice. Both global and adrenocortical‐specific deletion of the TP receptor lead to increased adiposity in mice by elevating corticosterone synthesis. Mechanistically, the TP receptor deletion increases the phosphorylation of steroidogenic acute regulatory protein (StAR) and corticosterone synthesis in adrenal cortical cells by suppressing p‐p38‐mediated phosphorylation of 14‐3‐3γ adapter protein at S71. The activation of the p38 in the adrenal cortical cells by forced expression of the MKK6EE gene attenuates hypercortisolism in TP‐deficient mice. These observations suggest that the TxA_2_/TP signaling regulates adrenal corticosterone homeostasis independent of the hypothalamic–pituitary–adrenal axis and the TP receptor may serve as a promising therapeutic target for hypercortisolism.

## Graphical abstract

Glucocorticoids are essential steroid hormones produced from the cortex of adrenal glands and play a pivotal role in various physiological processes. This study reveals that the TP receptor‐a regulator of platelet activation, modulates endogenous corticosterone biosynthesis via p‐p38/14‐3‐3γ/p‐StAR signaling in the adrenal glands. Therefore, the TP‐mediated pathway may be an attractive therapeutic target for the treatment of hypercortisolism.

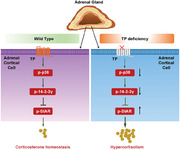

## 1 Introduction

Glucocorticoids (known as cortisol in humans and primates, and corticosterone in rodents) are essential steroid hormones that regulate various physiological processes such as development and metabolism.^[^ ^1^ ^]^ Abnormal glucocorticoid levels can lead to serious metabolic dysfunctions, such as Cushing's syndrome caused by excess glucocorticoid activity. This potentially lethal disorder is associated with significant metabolic comorbidities such as central obesity, insulin resistance, and diabetes.^[^ ^2^ ^]^ Upon activation of the hypothalamic–pituitary–adrenal (HPA) axis, corticotropin‐releasing hormone (CRH) and arginine vasopressin (AVP) are released from the hypothalamic paraventricular nucleus (PVN). Both CRH and AVP are transported to the pituitary gland, where they act synergistically to stimulate the secretion of adrenocorticotropic hormones (ACTHs). These hormones are then transported to the cortex of the adrenal gland, where they rapidly stimulate the biosynthesis and secretion of glucocorticoids.^[^ ^3^ ^]^ The HPA axis is also subjected to negative feedback inhibition, in which glucocorticoids inhibit their release by blocking the synthesis of ACTH and CRH in the pituitary gland and hypothalamus, respectively.^[^ ^4^ ^]^ The synthesis of corticosterone from cholesterol requires a series of reactions involving cytochrome P450 (CYP) enzymes. The rate‐limiting step in corticosterone synthesis is the translocation of cytosolic cholesterol from the outer to the inner mitochondrial membrane, which is governed by the steroidogenic acute regulatory protein (StAR).^[^ ^5^ ^]^ Multiple signaling pathways regulate StAR expression, activation, and/or degradation, such as cAMP/protein kinase A (PKA), PKC,^[^ ^6^ ^]^ and p38 MAPK signaling.^[^ ^7^ ^]^ However, the precise regulatory mechanism of the StAR function in adrenal steroidogenesis, particularly the local regulation in the adrenal cortex, is not fully understood.

Prostanoids are cyclooxygenase (COX) products derived from arachidonic acid, which include prostaglandin (PG) E_2_, PGD_2_, PGF_2α_, PGI_2_, and thromboxane (Tx) A_2_. All these prostanoids exert their biological functions via binding their specific G‐protein coupled receptors (EP1‐4, DP1‐2, FP, IP, and TP).^[^ ^8^ ^]^ Non‐steroid anti‐inflammatory drugs (NSAIDs), including aspirin and naproxen, exert antipyretic and analgesic effects primarily by reducing prostanoid synthesis through the inhibition of COX enzymes.^[^ ^9^ ^]^ Prostanoids have been reported to be expressed in the adrenal and regulate the HPA axis activity, as well as the secretion of ATCH and cortisol.^[^ ^10^ ^]^ For instance, PGE_2_ treatment reduces corticosteroid release, whereas PGF_2α_ increases corticosteroid release of interrenal and ovarian tissues in vitro.^[^ ^11^ ^]^ Interestingly, plasma TxB_2_, a stable metabolic product of TxA_2_, was substantially higher in patients with Cushing's syndrome.^[^ ^12^ ^]^ Elevated circulating ACTH is induced by mineral acids along with elevated plasma TxB_2_ levels in animals, which can be prevented by the TP antagonist, SQ‐29548.^[^ ^13^ ^]^ However, the involvement of the endogenous TxA_2_/TP signaling axis is involved in the regulation of adrenal corticosterone homeostasis is unknown.

In this study, we demonstrated that the global or adrenocortical‐specific deletion of TP increases adiposity in mice by elevating corticosterone production. Mechanistically, we identified a TP/p‐p38/14‐3‐3γ/p‐StAR signaling axis that mediated the regulation of endogenous corticosterone biosynthesis in the adrenal gland in mice. The activation of p38 MAPK in the adrenal cortex improved TP deficiency‐induced hypercortisolism and adiposity in mice. These findings uncover a novel role for the TxA_2_/TP axis in the regulation of corticosterone synthesis and adiposity.

## 2 Results and Discussion

### 2.1 TP Deficiency Leads to Hypercortisolemia in Mice

**Figure 1:**
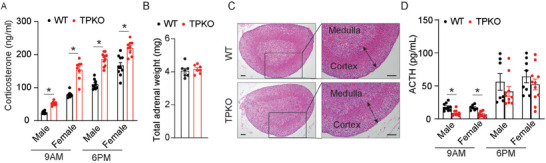
Deletion of TP increases corticosterone production in mice. A) Corticosterone levels in the plasma of WT and TPKO mice at indicated times (*n* = 7–8). B,C) Total adrenal weight (sum of left and right adrenals) (B) (*n* = 7) and representative hematoxylin/eosin (H&E)‐stained adrenal section images (C) of male WT and TPKO mice at 8 weeks of age. Scale bars, 100 µm. D) ACTH levels in the plasma of WT and TPKO mice (*n* = 7–11). For all panels, statistical significance was assessed by unpaired Student's *t‐test*. Data are presented as mean ± SEM and ^*^p < 0.05.

We first tested the distribution of TP in adrenal gland tissue by in situ hybridization (ISH). ISH analysis demonstrated that *TP* expression was highly expressed in the adrenal cortex (Figure S1A, Supporting Information), indicating TP may be involved in the regulation of corticosterone generation. Corticosterone secretion exhibits a robust daily rhythm.^[^ ^14^ ^]^ TP deletion enhanced plasma corticosterone levels in both male and female mice at different time points tested, without significantly influencing the corticosterone oscillation (**Figure** **1**A). However, TP ablation did not markedly affect adrenal gland weight and morphology in the mice (Figure 1B,C). The ACTH‐induced corticosterone production was also increased in TPKO mice, indicating that the HPA axis was not severely impaired in TPKO mice (Figure S1B, Supporting Information). Plasma ACTH levels in the TPKO mice tended to be declined (Figure 1D), probably due to feedback suppression. Taken together, the hypercortisolemia in TPKO mice may have resulted from enhanced activity in adrenal gland tissues.

### 2.2 TP Depletion Increases Adipogenesis in Mice

**Figure 2:**
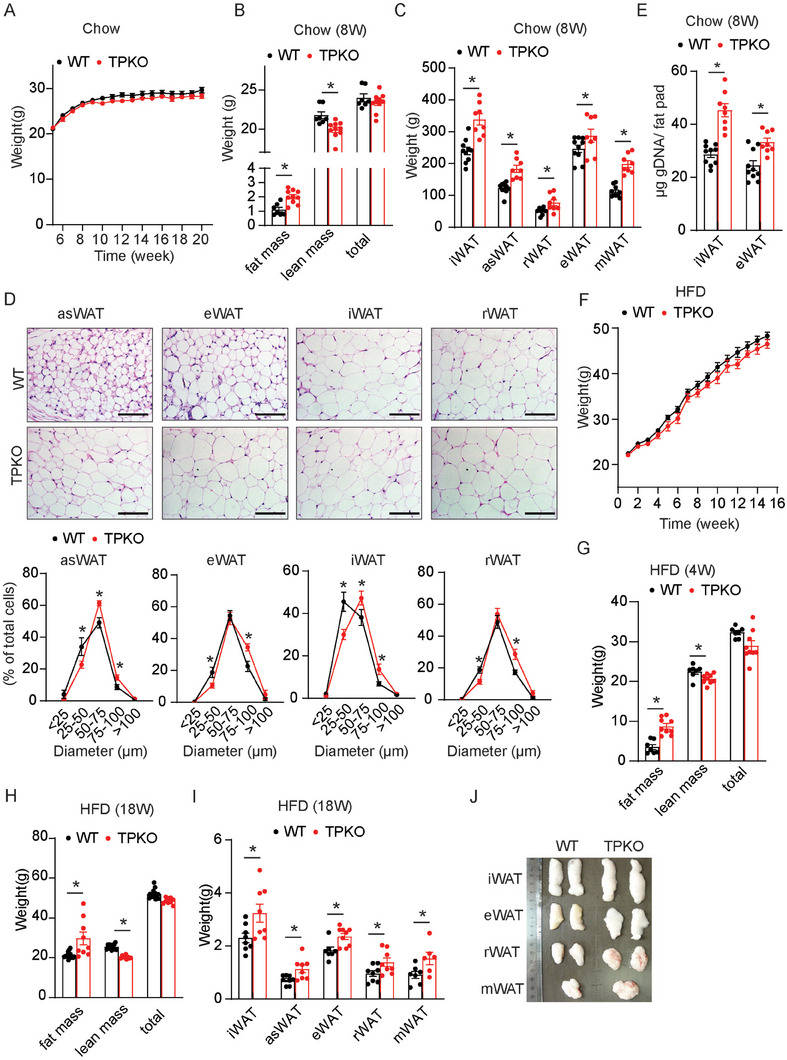
Increased adipogenesis in male TPKO mice. A–E) Analysis of metabolic parameters of male WT and TPKO mice on chow diet feeding, including A) body weight (*n* = 13–17); B) body composition of mice at 8 weeks (*n* = 7–10); C) fat mass weight of the indicated tissues in mice at 8 weeks (*n* = 8–10); D) H&E images and quantitative statistics on the proportion of adipocytes size of asWAT, eWAT, iWAT, and rWAT from mice at 8 weeks, scale bars, 100 µm (*n* = 7–10); E) total genomic DNA from iWAT and eWAT of mice at 8 weeks (*n* = 8–10). F–J) Analysis of metabolic parameters of male WT and TPKO mice on HFD feeding, including F) body weight (*n* = 15–21) and G,H) body composition (*n* = 7–9); I) fat mass weights (*n* = 6–9) and images J) of indicated tissues in mice after 18 weeks of HFD. Statistical significance was assessed by unpaired Student's *t‐test* (B–C, E, G–I), or *two‐way* ANOVA followed by Bonferroni's multiple comparisons test D). Data are presented as mean ± SEM and ^*^p < 0.05.

Chronic glucocorticoid exposure causes hyperplasia of the adipose tissue in both patients and rodents.^[^ ^15^ ^]^ Despite their overall normal weight (**Figure** **2**A), TPKO mice fed a chow diet showed markedly increased fat mass and decreased lean mass (Figure 2B). The fat pad weights of the inguinal (i), anterior subcutaneous (as), retroperitoneal (r), epididymal (e), and mesenteric (m) white adipose tissue (WAT) pads were higher in TPKO mice than in the control mice (Figure 2C). A gain in adipose tissue mass results from an increase in adipocyte cell size (hypertrophy), adipocyte cell number (hyperplasia), or both.^[^ ^16^ ^]^ Hematoxylin and eosin (H&E) staining showed that the size of adipocytes in the TPKO mice was dramatically increased (Figure 2D). Moreover, the iWAT and eWAT tissues contained more genomic DNA (gDNA) in TPKO mice than in control mice (Figure 2E), indicating adipocyte hyperplasia in TPKO mice.

To understand the role of TP in diet‐induced adipogenesis, we challenged mice with a high‐fat diet (HFD); TP deletion, without affecting overall body weight (Figure 2F), resulted in increased fat mass, and decreased lean mass in HFD‐fed mice (Figure 2G,H). Again, the major WAT pads were heavier in TPKO mice than in WT controls (Figure 2I,J).

To further determine whether the increased fat mass in TPKO mice was caused by higher corticosterone, we surgically removed bilateral adrenal glands from WT and TPKO male mice on postnatal day (P) 21 (Figure S2A, Supporting Information). Adrenalectomization (ADX) led to minimal levels of plasma corticosterone in mice and markedly attenuated the increased fat mass in TPKO mice without influencing total body weight (Figure S2B–D, Supporting Information). Overall, these results indicated that the enhanced accumulation of WAT can be attributed to hypoadrenalism in TPKO mice.

### 2.3 TP Deficiency in the Adrenal Cortex Increases Steroidogenesis and Adiposity in Mice

**Figure 3:**
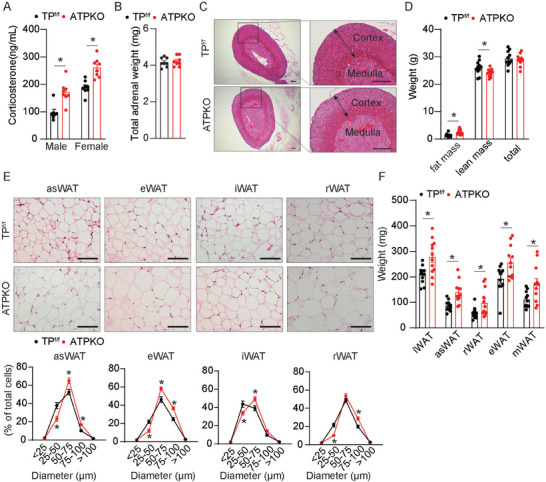
Adrenal cortical‐specific TP ablation promotes corticosterone synthesis and adiposity in mice. A) Analysis of plasma corticosterone levels of male TP^f/f^ and ATPKO mice on chow diet feeding (*n* = 7–8); B,C) Total adrenal weight (sum of left and right adrenals) (B) (*n* = 8) and representative H&E‐stained adrenal section images C) of male TP^f/f^ and ATPKO mice. Scale bars, 100 µm; D–F) (D) body composition (*n* = 11–12), E) macroscopic images quantitative statistics (*n* = 10), and F) fat mass weight (*n* = 11–12) on the proportion of indicated tissues in male mice at 8 weeks of age. Scale bars, 100 µm. Statistical significance was assessed by unpaired Student's *t‐test* (A, C, and E). Data are presented as mean ± SEM. ^*^p < 0.05 versus TP^f/f^.

To further investigate the role of the TxA_2_/TP axis in adrenal corticosterone synthesis, adrenal cortical‐specific TP‐deficient mice were generated by crossing TP^flox/flox^ (TP^f/f^) mice with akr1b7‐Cre mice (Figure S3A,B, Supporting Information) (called ATPKO). The TP gene was specifically deleted in the adrenal gland (Figure S3C, Supporting Information), but not in gonadal glands and other tissues (Figure S3D,E, Supporting Information). Furthermore, plasma corticosterone levels were elevated in ATPKO mice with normal adrenal gland weight and morphology (**Figure** **3**A–C). Consistently, TP deletion specifically in the adrenal glands increased fat mass in male mice (Figure 3D–F).

### 2.4 TP Ablation Increases Phosphorylation of StAR in the Adrenal Gland Tissue

**Figure 4:**
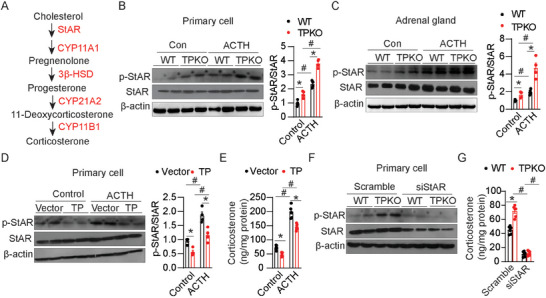
TP deficiency increases the phosphorylation of StAR in the adrenal gland. A) A schematic presentation of corticosterone synthesis pathways in mice. B,C) Western blot analysis and quantitation of indicated proteins in primary adrenal cortical cells (B) and adrenal glands (C) from WT and TPKO mice treated with or without ACTH (*n* = 4). D,E) Western blot analysis and quantitation of indicated proteins in expression (D) (*n* = 4) and corticosterone production (E) (*n* = 6) in WT primary adrenal cortical cells infected with GFP or TP overexpression lentivirus, then treated with or without ACTH. F,G) Western blot analysis of indicated proteins expression (F) and corticosterone production (G) (*n* = 6) in cultured WT and TP KO primary adrenal cortical cells infected with scramble or StAR knockdown lentivirus. Statistical significance was assessed by *two‐way* ANOVA (B–G). Data are presented as mean ± SEM. ^*^p < 0.05 versus WT; *^#^p* < 0.05 versus Control.

Glucocorticoid biosynthesis depends on the continuous ACTH stimulation of adrenal steroidogenic and detoxification genes, through the cAMP/PKA signaling pathway.^[^ ^17^ ^]^ Therefore, we studied the expression levels of ACTH‐dependent (*StAR*, *Akr1b7*, *Cyp11a1*, and *Cyp11b1*) and ‐independent (*Cyp11b2*) genes in the adrenal glands of TPKO and WT mice (**Figure** **4**A). Surprisingly, the mRNA expression of these steroidogenic genes was unaltered in the TPKO adrenal glands (Figure S4, Supporting Information). In contrast, the levels of phosphorylated StAR (p‐StAR) were increased in the primary TPKO adrenal cortical cells with and without ACTH treatment (Figure 4B). Moreover, we also observed the elevated phosphorylation of StAR protein in TPKO adrenal gland tissues compared to that in WT controls (Figure 4C). In cultured adrenal cortical cells, TP overexpression dramatically decreased phosphorylation of StAR and corticosterone production under both basal and ACTH‐treatment conditions (Figure 4D,E). Furthermore, StAR knockdown in adrenal cortical cells abrogated the TP deficiency‐induced elevation of corticosterone production (Figure 4F,G). However, knockout or overexpression of TP did not affect StAR abundance either in cultured primary adrenal cortical cells or in mouse adrenal glands (Figure 4B–F), indicating that TP ablation promotes corticosterone production via the regulation of StAR phosphorylation.

### 2.5 TP Deficiency Increases Corticosterone Production in Adrenal Cortical Cells Through Suppression of p38 MAPK

**Figure 5:**
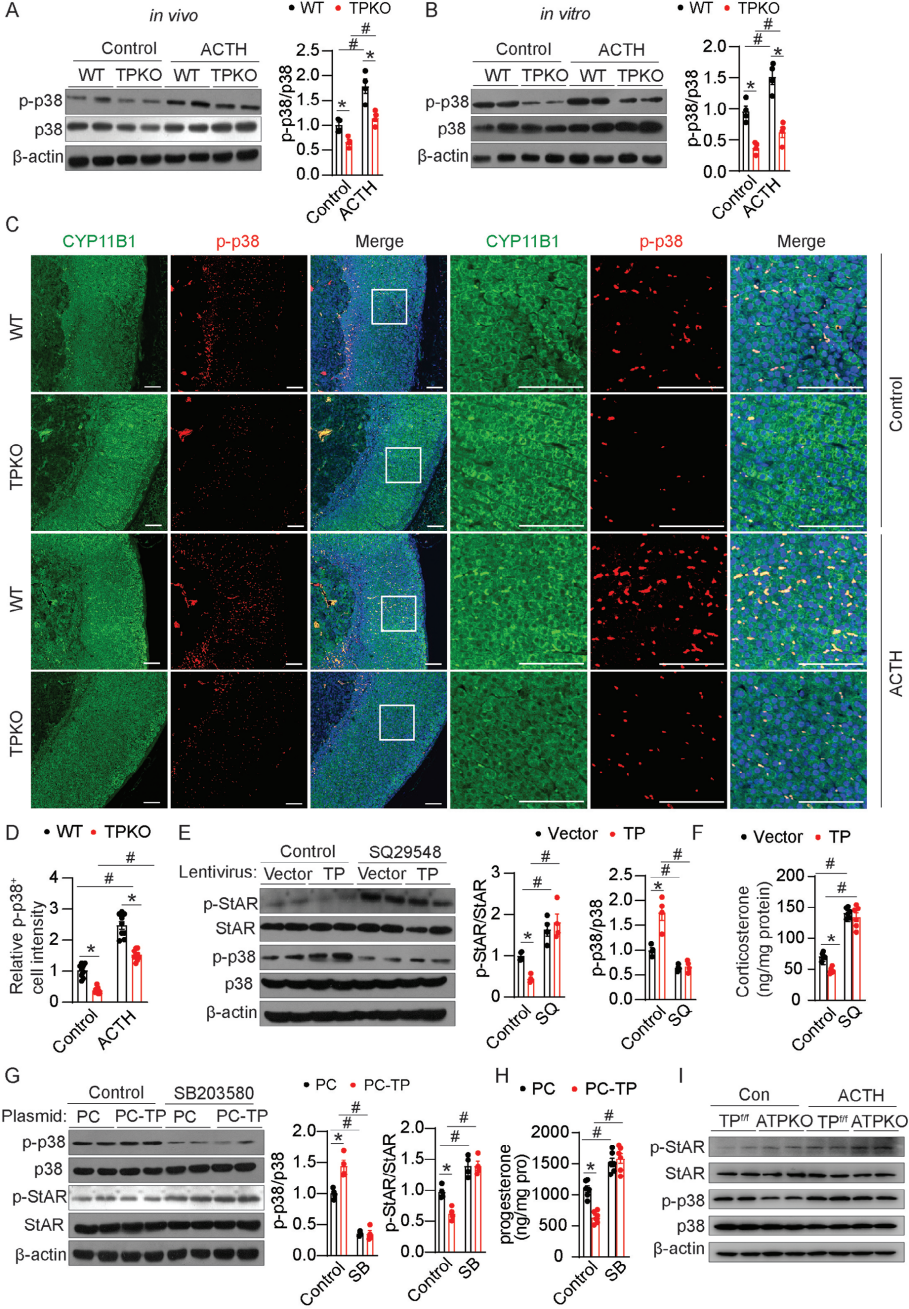
TP deficiency increases p‐StAR through the inhibition of p38. A,B) Western blot analysis and quantitation of indicated proteins in adrenal glands (A) and primary adrenal cortical cells (B) from WT and TPKO mice treated with or without ACTH (*n* = 4). C,D) Immunofluorescence analysis of CYP11B1 and p‐p38 in adrenal glands of WT and TPKO mice treated with or without ACTH (C), and quantification of the percentage of CYP11B1 positive cells expressing p‐p38 (D) (*n* = 9). Scale bars, 50 µm. E,F) Western blot analysis and quantitation of indicated proteins (E) (*n* = 4) and corticosterone production (F) (*n* = 6) in primary adrenal cortical cells infected with GFP or TP lentivirus, then treated with or without SQ29548. G, H) Western blot analysis and quantitation of indicated proteins (G) (*n* = 4) and progesterone production (H) (*n* = 6) in Y1 cells infected with control or TP overexpression plasmid, then treated with or without SB203580. I) Western blot analysis of indicated proteins in adrenal glands from male WT and ATPKO mice. Statistical significance was assessed by *two‐way* ANOVA. Data are presented as mean ± SEM. ^*^p < 0.05 versus WT; *^#^p* < 0.05 versus Control.

Activation of p38 MAPK is associated with the downregulation of StAR phosphorylation and steroid synthesis.^[^ ^18^ ^]^ Immunoblot analysis revealed that TP disruption dramatically decreased the phosphorylation of p38 in both mouse adrenal gland tissues and primary cultured adrenal cortical cells (**Figure** **5**A,B). In addition, ACTH‐induced p38 phosphorylation was also attenuated by TP deletion in the adrenal cortical cells (Figure 5A,B). 11β‐hydroxylase (CYP11B1) was used to label the *zona fasciculata* (ZF) zone of the adrenal gland, which mainly produces corticosterone. Double immunostaining of p‐p38 and CYP11B1 confirmed that p‐p38 positive cells in the ZF zone were substantially fewer in TPKO mice with and without ACTH treatment than in WT mice (Figure 5C,D). TP ablation did not affect the area of the ZF zone (CYP11B1^+^ cells) in mice (Figure 5C). In contrast, TP overexpression dramatically increased p38 phosphorylation and suppressed StAR phosphorylation and corticosterone production in adrenal cortical cells, which were abolished by SQ29548‐the selective TP inhibitor (Figure 5E,F). The effect of the TP activation on the p‐p38/StAR signaling pathway was also evaluated in adrenocortical Y1 cells, which lack CYP21A2 expression and produce progesterone upon stimulation.^[^ ^19^ ^]^ Moreover, TP overexpression substantially increased p38 phosphorylation and decreased StAR phosphorylation and progesterone production in adrenocortical Y1 cells (Figure 5G,H). These effects were reversed by treatment with the p‐p38 inhibitor SB203580 (Figure 5G,H). Consistently, the reduced p38 activity and increased StAR phosphorylation were observed in adrenal gland tissues from ATPKO mice (Figure 5I).

**Figure 6:**
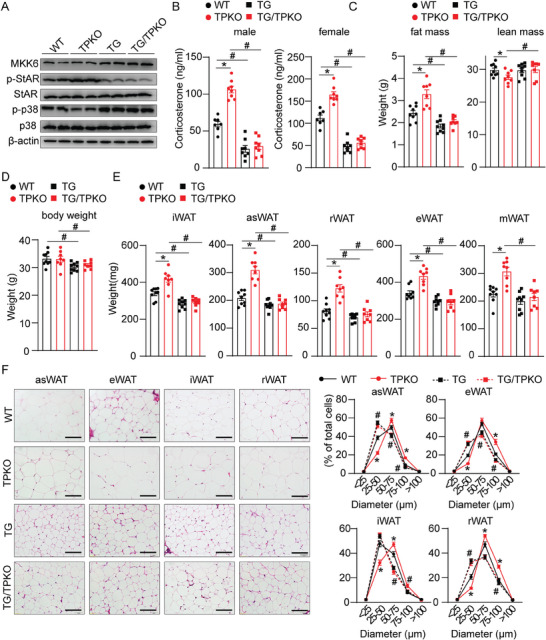
Adrenal cortical‐specific MKK6EE overexpression blunts hypercortisolism and adiposity in TPKO mice. A–F) Analysis of metabolic parameters of WT, TPKO, TG, and TG/TPKO mice on chow diet feeding (*n* = 7–9), including (A) western blot analysis of indicated proteins in adrenal glands from indicated mice; (B) plasma corticosterone levels of 9 AM; C) body composition; D) body weight; E) fat mass weight; and F) macroscopic images and quantitative statistics on the proportion of indicated tissues in male mice at 8 weeks of age, Scale bars, 50 µm. Statistical significance was assessed by *two‐way* ANOVA (B–E). Data are presented as mean ± SEM. ^*^p < 0.05 versus WT.

To further evaluate the role of the adrenal p‐p38 MAPK pathway in adrenal steroidogenesis in vivo, we generated a constitutively activated p38 transgenic mice (TG, Figure S5A, Supporting Information) in which a constitutively active mutant of *mitogen‐activated protein kinase kinase 6* allele (MKK6EE, a mutant MAPK kinase that specifically activates p38.^[^ ^20^ ^]^) was under the control of the 0.5‐kb aldo‐keto reductase 1B7 (akr1b7) promoter.^[^ ^21^ ^]^ Two transgenic lines with robust MKK6 expression and p38 phosphorylation in the adrenal cortex were identified (Figure S5B–D, Supporting Information). Conversely, forced activation of p38 MAPK by MKK6EE overexpression in the adrenal cortex decreased StAR phosphorylation in the adrenal gland tissues and serum corticosterone production in mice (Figure S5D,E, Supporting Information). Immunofluorescence staining also showed MMK6 was highly expressed in the adrenal cortex in MKK6EE adrenal cortical‐specific overexpression mice (Figure S5F, Supporting Information). To further investigate the effect of p38 activation on adrenal steroidogenesis, we crossed the MKK6EE adrenal cortical‐specific overexpression mice (TG‐1) with TPKO mice. As shown in **Figure** **6**A–F, overexpression of MKK6EE in the adrenal cortex attenuated the increased StAR phosphorylation in the adrenal glands, suppressed the induction of plasma corticosterone, and reduced the exaggerated fat masses in TPKO mice. Collectively, these observations suggested that TP suppresses StAR phosphorylation and steroidogenesis via p38 activation.

TP receptor typically mediates downstream signaling through coupling Gαq.^[^ ^22^ ^]^ Gαq activates p38 through Ca^2+^‐dependent CAMKII signaling.^[^ ^23^ ^]^ A rapid increase of intracellular calcium was observed in Y1 cells upon TP agonist U46619 stimulation (Figure S6A, Supporting Information). We examined the expression levels of CaMKII isoforms (α, β, γ, and δ), and found CaMKIIγ isoform was highly expressed in the adrenal gland tissue in mice (Figure S6B, Supporting Information). As anticipated, the levels of p‐CAMKIIγ and p‐p38 were increased in Y1 cells by U46619 treatment, which was attenuated by either the phospholipase C inhibitor U73122 or the CaMKIIγ inhibitor KN93 (Figure S6C, Supporting Information). These data demonstrate that TP regulates p‐p38 through the Gq/CAMKIIγ pathway in adrenal cortical cells.

### 2.6 TP Activation Suppresses StAR Phosphorylation in Adrenal Cortical Cells Through p38/14‐3‐3γ Signaling Pathway

**Figure 7:**
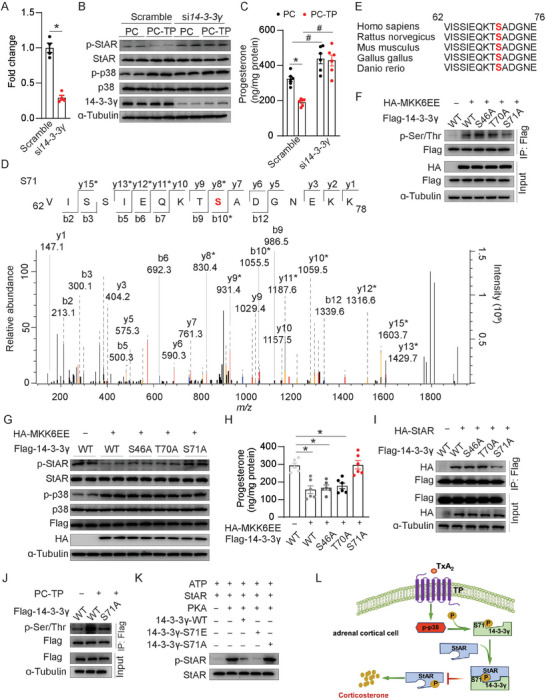
TP decreases corticosterone synthesis via the p‐p38/14‐3‐3γ/p‐StAR signaling pathway. A–C) (A) mRNA levels of 14‐3‐3γ (*n* = 4), and (B) western analysis indicated proteins, (C) progesterone production (*n* = 6) in control and TP overexpressing Y1 cells treated with scramble and si*14‐3‐3γ*. D) Mass spectrum showing that 14‐3‐3γ was phosphorylated at S71 by p38. E) Alignment of conserved S71 residues in 14‐3‐3γ protein sequences of different species. F–H) Co‐immunoprecipitation and western analysis indicated proteins (F and G) and progesterone production (H) (*n* = 6) in Y1 cells transfected with indicated plasmids. I,J) Co‐immunoprecipitation and western analysis indicated proteins in Y1 cells infected with indicated plasmids. K) Western analysis indicated proteins of in vitro kinase assay of recombinant WT and mutant (S71E and S71A) 14‐3‐3γ proteins. L) Model depicting the function of TP in regulating corticosterone synthesis. Statistical significance was assessed by unpaired Student's *t‐test* (A and H) or *two‐way* ANOVA (C). Data are presented as mean ± SEM. ^*^p < 0.05 versus PC; *^#^p* < 0.05 versus Scramble.

It has been suggested that adaptor protein 14‐3‐3γ negatively regulates steroidogenesis by interacting with StAR.^[^ ^24^ ^]^ We first determined whether 14‐3‐3γ was involved in the TP‐mediated suppression of steroidogenesis by employing the siRNA strategy (**Figure** **7**A). Overexpression of exogenous TP markedly increased p38 phosphorylation, diminished StAR phosphorylation, and decreased progesterone production in Y1 cells (Figure 7B,C). However, the knockdown of *14‐3‐3γ* abolished the TP‐mediated suppression of StAR phosphorylation and progesterone production (Figure 7B,C), indicating that 14‐3‐3γ is required for TP‐mediated suppression of steroidogenesis. Of note, the phosphorylation of p38 and total expression of p38 was not affected in Y1 cells by the 14‐3‐3γ knockdown (Figure 7B), implicating that 14‐3‐3γ may function as p‐p38 downstream.

Next, we explored whether p38 directly phosphorylates 14‐3‐3γ in in vitro system. Mass spectrometry analysis of 14‐3‐3γ phosphorylation identified three residues, including S46, T70, and S71 in 14‐3‐3γ that were phosphorylated by p‐p38 in vitro (Figure 7D; Figure S7A,B, Supporting Information). Alignment of the 14‐3‐3γ amino acid sequences of different species suggested that T70 and S71 are highly conserved in vertebrates (Figure 7E). We further constructed three phosphorylation‐deficient 14‐3‐3γ mutants by replacing serine (S) or tyrosine (T) with alanine (A) (Figure 7F). Mutant S71A markedly attenuated p38 mediated14‐3‐3γ phosphorylation in adrenal cortical cells, whereas S46A or T70A didn't (Figure 7F), indicating p38 phosphorylates 14‐3‐3γ predominantly at S71 residue in adrenal cortical cells. Moreover, S71A mutation, not S46A or T70A, reversed 14‐3‐3γ overexpression‐mediated suppression of StAR phosphorylation and progesterone production in Y1 cells (Figure 7G,H). The direct binding of 14‐3‐3γ with StAR was much weakened by phosphorylation‐deficient S71A mutation in Y1 cells (Figure 7I), suggesting p38 inhibits StAR activity through the enhancement of 14‐3‐3γ phosphorylation‐mediated binding with StAR. In addition, TP overexpression increased the phosphorylation of 14‐3‐3γ in Y1 cells, which was attenuated by the S71A 14‐3‐3γ mutant (Figure 7J). Finally, in vitro kinase assay further demonstrated that PKA‐induced phosphorylation of StAR was markedly decreased by the addition of 14‐3‐3γ protein. The S71E mutation of 14‐3‐3γ to mimic p38‐mediated phosphorylation further inhibited PKA‐induced phosphorylation of StAR (Figure 7K). In contrast, phosphorylation‐resistant mutation S71A of 14‐3‐3γ did not notably influence PKA‐induced phosphorylation of StAR (Figure 7K). Thus, TP activation suppresses PKA‐mediated phosphorylation of StAR in adrenal cortical cells through increasing scaffolding protein14‐3‐3γ binding to StAR by p38 (Figure 7L).

## 3 Conclusion

**Figure 8:**
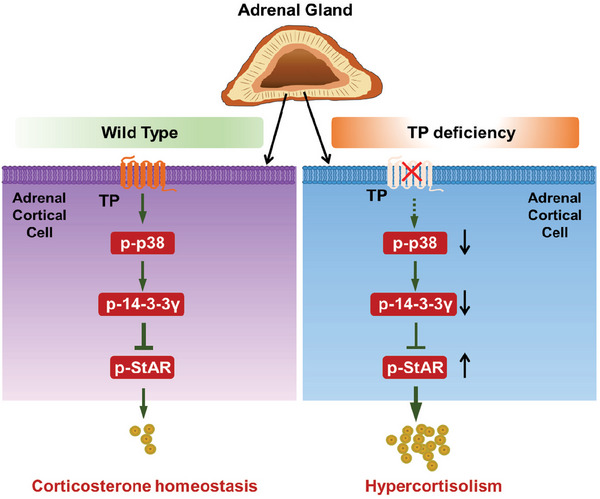
Diagram for TP‐mediated signal pathway in the regulation of corticosterone homeostasis in the adrenal cortex. Under normal conditions, TP activation represses the excessive production of corticosterone in the adrenal cortex through p38/14‐3‐3γ/p‐StAR signaling (left). In the absence of the TP receptor, corticosterone production is in the adrenal cortex enhanced by activation of StAR via suppressing the p‐38 inhibitory activity (right).

Steroidogenic processes are typically controlled by trophic hormones, but also are subject to intrinsic post‐transcriptional and post‐translational pathways.^[^ ^25^ ^]^ In this study, we observed that TP was predominantly expressed in the adrenal gland tissues of mice. Global or adrenal cortex‐specific deletion of TP increased adiposity in mice by increasing adrenal corticosterone production through the p‐p38/14‐3‐3γ/p‐StAR pathway, suggesting TP is implicated in intrinsic regulation of corticosterone production (**Figure** **8**). Therefore, TP and its downstream pathway may constitute an attractive target for the treatment of patients with hypercortisolism.

TxA_2_, known as a vasoconstrictor and platelet activator, also plays important roles in various pathophysiological processes such as allergy, inflammation, and carcinogenesis.^[^ ^26^ ^]^ The TxA_2_/TP axis in white adipose tissue is activated and mediates insulin resistance in obese mice.^[^ ^27^ ^]^ Interestingly, TxA_2_ restrains VEGF‐induced endothelial cell differentiation and PGI_2_‐induced brown adipocyte differentiation of adipose‐derived stem cells.^[^ ^28^ ^]^ Moreover, the TxA_2_ stable analog U‐46619 increases ACTH and corticosterone levels in the fetal plasma and brain.^[^ ^29^ ^]^ The TP receptor is abundantly expressed in the adrenal cortex, and the ablation of TP in adrenocortical cells led to hyperadrenocorticism and adiposity in mice, indicating that the TxA_2_/TP axis is involved in corticosterone homeostasis. However, we did not detect any increase in serum ACTH levels in TPKO mice, indicating that TP‐regulated adrenal corticosterone synthesis is independent of ACTH.

The StAR protein regulates the rate‐limiting step in steroid biosynthesis, that is, the delivery of cholesterol from the outer to the inner mitochondrial membrane. The StAR protein is initially synthesized as a 37 kDa protein and is then converted to a 30 kDa form in the mitochondria, and it is fully activated by phosphorylation of Ser194 mediated by protein kinase A.^[^ ^30^ ^]^ Accumulating evidence has shown that inflammatory microenvironments including prostanoids play important roles in the regulation of sterodiogenesis.^[^ ^31^ ^]^ In this study, activation of the TxA_2_/TP axis suppresses StAR phosphorylation in both primary adrenal cortical cells and Y1 cells, subsequently inhibiting corticosterone secretion. Consistent with our observations, TP antagonists have been shown to increase StAR‐mediated steroid production in mouse Leydig cells.^[^ ^32^ ^]^ Similarly, PGF_2α_ exerts its anti‐steroidogenic effect via decreasing StAR mRNA levels.^[^ ^33^ ^]^ In contrast, PGE_2_ mediates ACTH‐induced corticosterone release by increasing StAR expression in rats.^[^ ^34^ ^]^

p38 kinases are structurally conserved proline‐directed serine/threonine kinases of the mitogen‐activated protein kinase (MAPK) family. Unlike prototypic MAPKs ERK1 and ERK2, p38 kinases are generally activated by pro‐inflammatory signals and environmental stresses, but not by mitogens.^[^ ^35^ ^]^ The p38 MAPK‐mediated signaling pathway is involved in both oxidative stress and aging‐triggered suppression of adrenal steroidogenesis.^[^ ^36^ ^]^ Ablation of p38α expressed isoform in adrenocortical cells increases corticosterone levels in mice by increasing StAR activity.^[^ ^18b^ ^]^ We found that TP activation restrained adrenal steroidogenesis via the p38‐StAR pathway and that this regulation was independent of the HPA axis. Interestingly, the p38‐mediated inhibition of steroidogenesis in H_2_O_2_‐treated Y1 cells may be also associated with reduced CREB transcriptional activity.^[^ ^37^ ^]^ Interestingly, MKK6 deletion promotes adipose tissue browning and increases organismal energy expenditure in mice by increasing UCP1 expression.^[^ ^38^ ^]^ Since low Akr1b7 expression is detected in adipose tissues, forced expression of active p38 driven by the Akr1b7 promoter in the MKK6EE transgenic mice may directly influence adipose tissue browning in addition to suppressing corticosterone secretion.^[^ ^39^ ^]^

The 14‐3‐3 protein family consists of seven members with highly conserved eukaryotic scaffolding/adaptor proteins and participates in many cellular processes, such as signal transduction, energy metabolism, and protein trafficking.^[^ ^40^ ^]^ Among them, 14‐3‐3γ is widely expressed and modulates various physiological processes including steroidogenesis through specific phosphopeptide motifs‐mediated protein‐protein interactions.^[^ ^41^ ^]^ In hormone‐dependent steroidogenic MA‐10 Leydig cells, 14‐3‐3γ binds StAR on S194 to block StAR activity that negatively regulates steroidogenesis.^[^ ^24^ ^]^ Here, we identified a functional p38‐mediated phosphorylation residue S71 of 14‐3‐3γ, which is highly conserved in vertebrates. It was shown that TP activation promotes the 14‐3‐3γ phosphorylation at S71 by p38 and subsequently increases its inhibitory binding with StAR to suppress steroidogenesis. In addition, 14‐3‐3γ phosphorylation at residue S58 tightens StAR binding and inhibits steroidogenesis in MA‐10 Leydig cells.^[^ ^42^ ^]^

In summary, we identified that the TP receptor regulates adrenal corticosterone homeostasis through the p38/14‐3‐3γ/p‐StAR signaling pathway, suggesting that intervention of the TP‐mediated pathway may be a promising strategy for the treatment of hypercortisolism.

## 4 Experimental Section

### Animals

All animal experiments were approved by the Institutional Animal Care and Use Committees of the Tianjin Medical University (Approval No: TMUaMEC 2 020020). All mice were maintained on a C57BL/6 genetic background. The TPKO and TP‐floxed (Jax: 02 1985) mice were genotyped and maintained as previously described.^[^ ^28^, ^43^ ^]^ Mice were maintained on a chow diet (Harlan Teklad, 8664) under a virus‐free, 12 h light/dark cycle at 23 °C for standard room temperature housing. Eight‐week‐old mice were fed a 60% high‐fat diet (D12492, Research Diets) at room temperature and their body weights were measured weekly.

### Generation of Mice

The akr1b7‐Cre mice were generated by CRISPR‐cas9‐mediated genome editing (Cyagen Biosciences). The cas9‐guide RNA (gRNA) target sequences (gRNA1: GGCAGGCTTAAAGGCTAACC; gRNA2: CTCCAGTCTTTCTAGAAGAT) were designed in the regions of intron 1 of ROSA26 in a reverse orientation to promote DNA breaks and homologous recombination. The “Akr1b7 promoter‐Kozak‐iCre‐rBG pA‐Intragenic region” cassette in the donor oligo was designed and synthesized.^[^ ^21^ ^]^ Then, cas9 mRNA and gRNA generated by in vitro transcription and donor oligo were co‐injected into fertilized mouse eggs. The pups were identified by PCR followed by sequence analysis using the following primers (WT‐F: CACTTGCTCTCCCAAAGTCGCTCA; WT‐R: ATACTCCGAGGCGGATCACAA; Cre‐F: CAGCCCTCCTATTGCATCGTAA; Cre‐R: CATAGAAAAGCCTTGACTTGAGGT).

MKK6‐TG mice were generated using the 0.5 akr1b7‐CAT plasmid with slight modifications (Cyagen Biosciences), which comprises a 0.5‐kb fragment (−510/+44) of the 5′‐flanking sequence and a 3.5‐kb intragenic segment of the akr1b7 gene, spanning intron 1 to intron 2.^[^ ^36a^ ^]^ The Cre gene was replaced by the MKK6 mutant cDNA.^[^ ^44^ ^]^ The vector was linearized by HindIII/SacI digestion, and the purified product was introduced into C57BL/6 ES cells. Correctly targeted ES cell clones were identified by the long‐range PCR screening. The following primers are used for genotyping: forward 5′‐ CAGGCATTTCATCTGCTCACTCA −3′, reverse 5′‐ CTTCACCTCAAAGTTCTGATTTCCA −3′.

### Adrenalectomy

Bilateral adrenalectomies were performed as previously described.^[^ ^45^ ^]^ The mice were fully anesthetized with 1–1.5% isoflurane gas and buprenorphine (Temsgesic, 0.003 mg/100 g body weight) was administered subcutaneously as an analgesic. A 0.5 cm skin incision was made on the back side of the mouse, then the skin was separated, and a 2 mm muscle incision was made on the top of each adrenal gland. The entire adrenal gland was removed using a pair of sterile fine‐curved forceps. The incision was closed with surgical staples, and 0.8 mL of 0.9% sterile saline solution was injected subcutaneously to restore body fluids. The mice were placed in individual clean cages and allowed to recover under a heating lamp. After recovery, the mice were provided food and drinking water containing 0.9% sodium chloride ad *libitum*. Control mice (sham) underwent the same surgical procedure as the ADX group, except that their adrenal glands were not excised.

### Isolation and Differentiation of Adrenal Cortical Cells

Adrenal cortical cells were isolated and differentiated as previously described with slight modifications.^[^ ^46^ ^]^ Adrenal glands from ten mice (age 6–10 weeks, of both sexes) per experiment were excised and placed in dishes with ice‐cold PBS. The fat tissue surrounding the adrenals was carefully removed and then the adrenal cortex was thoroughly isolated from the medulla. All cortical tissues were pooled, pelleted (350 g, 5 min), and digested for 20 min at 37 °C while shaking (1.8 mg mL^−1^ collagenase, 10 mg mL^−1^ BSA, 0.18 mg mL^−1^ DNAse in PBS; Sigma–Aldrich). The digestion was stopped by washing it twice in PBS and the adrenocortical cells were resuspended in 1 mL Dulbecco's modified Eagle medium (DMEM/F12)–high glucose (Thermo Fisher Scientific) containing 10% steroid‐free fetal bovine serum (charcoal/dextran treated; Hyclone Laboratories), 1% antibiotic‐antimycotic solution (Thermo Fisher Scientific), 1% L‐Glutamine (Thermo Fisher Scientific) and 20 ng mL^−1^ basic fibroblast growth factor (bFGF) (Sigma–Aldrich). Adrenal cortical cells were cultured in Neurobasal Medium (Thermo Fisher Scientific) containing 2% B27, 1% antibiotic‐antimycotic solution, 1% L‐glutamine, and 20 ng mL^−1^ basic bFGF. The isolated cells were cultured in ultra‐low‐attachment surface plates (Corning) at 37 °C in a humidified atmosphere (95% O_2_, 5% CO_2_).

To differentiate the adrenal cells in vitro, spheres (after 6 days of proliferation), were plated into 24‐well plates (Corning) or 8‐well chamber plates (Ibidi) coated with 1 mg mL^−1^ poly‐D‐lysine (Merck Millipore) and 3 µL mL^−1^ bFibronectin (R&D Systems) and cultured in the absence of bFGF. The medium was replaced with fresh medium every 2–3 d.

### Cell Culture

Y‐1 cells (ATCC) were cultured in RPMI1640 (Thermo Fisher Scientific) supplemented with 10% FBS and 1% penicillin/streptomycin. Cells were maintained at 37 °C in a humidified atmosphere with 5% CO_2_.

HEK293‐T cells (ATCC) were cultured in Dulbecco's modified DMEM medium (Thermo Fisher Scientific) supplemented with 10% FBS and 1% penicillin/streptomycin. Cells were maintained at 37 °C in a humidified atmosphere with 5% CO_2_.

All cell lines were authenticated using the short tandem repeat method and were tested negative for mycoplasma.

### Body Composition

Body composition was used to determine the lean mass and fat mass values which were obtained with Echo MRI (Echo Medical Systems, Houston, Texas) using a 3‐in‐1 Echo MRI Composition Analyzer.

### Assay of Corticosterone, Progesterone, and ACTH

Plasma was isolated from mouse blood and immediately frozen at −80 °C until assay. The concentrations of corticosterone, progesterone, and ACTH in the plasma or culture medium were determined using an ELISA kit (Enzo Life Sciences for progesterone and corticosterone, Abcam for ACTH) according to the manufacturer's protocol.

### ACTH Administration

Mice were injected intraperitoneally with 10 µg kg^−1^ ACTH (1–24) (Sigma–Aldrich). Blood samples were collected 3 h after administration.

### Genomic DNA Content Analysis

Total genomic DNA was obtained from the whole iWAT and eWAT deposits harvested from the indicated mice using a DNeasy Blood and Tissue Kit (Qiagen) and quantified using a Nanodrop 2000c (Thermo Fisher Scientific).

### Histological Analysis

Adrenal glands from male mice (8–10 weeks old) were fixed overnight in Z‐fix (buffered zinc formalin fixative) and embedded in paraffin. Paraffin blocks were sectioned into 5 µm slices and stained with Hematoxylin and Eosin (H&E).

Fat tissues collected from mice were immediately fixed in 4% formaldehyde, incubated overnight at 4 °C, and washed with 70% ethanol. Formalin‐fixed, paraffin‐embedded tissues were stained with H&E. Adipocyte size was calculated using Image J as previously described.^[^ ^47^ ^]^

### Immunofluorescence Microscopy of Mouse Adrenal Gland

Sections of the adrenal glands were deparaffinized, rehydrated, washed in PBS, and blocked with Dako Protein block for 30 min at room temperature. Antigen retrieval was performed in a pressure cooker (Decloaking Chamber, Biocare Medical) in citrate buffer (pH 6.0) for CYP11B1 and p‐p38. Immunostaining was performed by incubating the sections overnight at 4 °C with antibodies in Dako antibody diluent. Alexa Fluor 488‐ or Alexa Fluor 594‐conjugated secondary antibodies were added for 1 h at room temperature (1:500, Thermo Fisher Scientific), and the nuclei were counterstained using the SlowFade Gold Anti‐fade reagent with DAPI (Vector).

### RNA Isolation/Quantitative RT‐PCR

Total RNA was extracted from the cells or tissues using a Direct‐zol RNA MiniPrep kit (ZYMO Research) or TRIzol reagent (Invitrogen). Reverse transcription was performed with 1 µg of total RNA using a High‐Capacity cDNA Reverse Transcription Kit (Thermo Fisher Scientific). qRT‐PCR was performed using an ABI PRISM 7500 instrument (Applied Biosystems). Melting curve analysis was performed to confirm the RT‐PCR products.

### Immunoprecipitation

The cells were collected and suspended in 700 µL lysis buffer (0.5% Nonidet P‐40, 5 mm EDTA, 5 mm EGTA in PBS) containing protease inhibitors and then passed 12 times through a 22G needle followed by centrifugation at 13 200 rpm for 10 min at 4 °C. Ninety microliters of the supernatant were mixed with membrane solubilization buffer plus the 4 × loading buffer as the input sample. Six hundred microliters of supernatants were immunoprecipitated with beads conjugated with indicated antibodies at 4 °C for 6–8 h. The beads were then washed with IP buffer (0.5% Nonidet P‐40, 5 mm EDTA, 5 mm EGTA in PBS) five times at 4 °C, then boiled with 2 × loading buffer (75 mm Tris‐HCl, pH 6.8, 50 mm NaCl, 6% SDS, 15% glycerol and 0.02% bromophenol blue) at 95 °C for 10 min and mixed up a solution of equal parts supernatants. Aliquots were analyzed by immunoblotting.

### In Vitro Kinase Assay

In brief, commercial StAR recombinant protein (0.5 µg) and cAMP‐dependent Protein Kinase (PKA), catalytic subunit (0.5 µg) was incubated with HEK293T‐purified WT or S71E, or S71A 14‐3‐3γ (0.5 µg) in kinase buffer (60 mm HEPES pH 7.5, 5 mm MgCl_2_, 5 mm MnCl_2_, 3 µm Na_3_VO_4_, and 1.25 mm DTT, 500 µm ATP) to a final volume of 90 µL at 37 °C for 30 min. The reaction was stopped with the addition of 30 µL 4 × loading buffer and boiled for 10 min. The phosphorylation of substrate proteins was analyzed by western blotting.

### Immunoblotting

For immunoblotting analyses, tissues and cells were lysed in RIPA buffer containing protease and phosphatase inhibitors (Thermo Fisher Scientific). Protein levels were quantified using a BCA protein assay kit (Thermo Fisher Scientific), and lysates containing an equal amount of protein were subjected to SDS‐PAGE and transferred to polyvinylidene fluoride (PVDF) membranes, followed by incubations with primary and secondary antibodies.

### Lentivirus‐Mediated Gene Transfer

Mouse TP, psPAX2, and pMD2.G were used for the lentiviral package. High‐titer lentivirus was packaged into 293T cells using a lipofectamine2000 transfection agent. Viral supernatants were collected and used to treat the adrenal cortical cell infections.

### In Situ Hybridization

A 540 bp fragment of the TP cDNA was amplified using the following primers: 5′‐ GCTGCCGCCT GTGCTACTTC‐3′; 5′‐ GCTGCCGCCT GTGCTACTTC‐3′; and subcloned in pGEM‐T easy vector (Progema). Antisense riboprobes were synthesized and labeled with digoxigenin (Sigma–Aldrich). Adrenals were fixed overnight in 4% paraformaldehyde, embedded in paraffin, and sectioned. Sections were treated for 15 min with proteinase K (3 mg mL^−1^) at room temperature and washed with glycine (2 mg mL^−1^), and then with PBS. Sections were fixed with 4% paraformaldehyde for 5 min and washed with PBS. Samples were incubated in a hybridization mix (50% formamide; 4 x SSC; 10% Dextran sulfate; 1 x Denhart's; Salmon sperm DNA 250 mg mL^−1^; tRNA 250 mg mL^−1^) for 1 h at 42 °C. A digoxygenin‐labeled probe was added to the hybridization mix and incubated overnight at 42 °C. Slides were then treated to a series of washes in 2 x SSC and 1 x SSC at 42 °C and 0.2 x SSC at room temperature. The sections were washed in buffer 1 (150 mm NaCl; 100 mm Tris, pH 7.5), blocked with Boehringer blocking reagent in buffer 1, and incubated for 1 h at room temperature with a peroxidase‐conjugated anti‐digoxygenin antibody. After several washes in buffer 2 (150 mm NaCl; 100 mm Tris, pH 9.5; 5 mm MgCl_2_), peroxidase activity was detected by incubation with 0.18 mg mL^−1^ BCIP and 0.34 mg mL^−1^ NBT in buffer 2. In situ hybridization slides were observed and photographed using an Axiophot microscope (Carl Zeiss, Zurich, Switzerland).

### Statistical Analyses

All statistical tests were performed using the GraphPad Prism 7 software (GraphPad Software). Data are presented as means ± SEM unless otherwise stated. Data from two groups were analyzed using an unpaired two‐tailed Student's *t*‐test. Data from the two groups and repeated measurements were analyzed using a two‐way ANOVA with Bonferroni's correction. Data comparing multiple groups with a single control group was analyzed using Bonferroni's multiple comparison test, and each cell mean was compared with the mean of the control. Significance is indicated as follows: ^*^p < 0.05. The presented histological images are representative of the biological replicates.

## Conflict of Interest

The authors declare no conflict of interest.

## Supporting information

Supporting Information

## Data Availability

The data that support the findings of this study are available from the corresponding author upon reasonable request.
