# Supplementary material for: Thromboxane A2 Modulates de novo Synthesis of Adrenal Corticosterone in Mice via p38/14‐3‐3γ/StAR Signaling
Source: Adv Sci (Weinh). 2024 Mar 9;11(18):2307926. doi: 10.1002/advs.202307926 (PMC11095200; doi:10.1002/advs.202307926)
Supplement: Supplementary file 1 — Supporting Information [file ADVS-11-2307926-s001.pdf]

ADVANCED SCIENCE
Open Access

Supporting Information

for *Adv. Sci.*, DOI 10.1002/advs.202307926

Thromboxane A_2 Modulates de novo Synthesis of Adrenal Corticosterone in Mice via p38/14-3-3γ/StAR Signaling

Shuai Yan, Yuanyang Wang, Bei Wang, Shengkai Zuo and Ying Yu**

Supporting Information

Thromboxane A_2 Modulates de novo Synthesis of Adrenal Corticosterone in Mice via p38/14-3-3γ/StAR Signaling

Shuai Yan, Yuanyang Wang, Bei Wang, Shengkai Zuo, Ying Yu**

Supplementary figures

Figure S1. TPKO mice displayed increased corticosterone production

Figure S2. Adrenalectomy decreased corticosterone levels in WT and TPKO mice

Figure S3. Generation of adrenal cortex-specific TP knockout mice

Figure S4. Expression of steroidogenic genes in adrenal glands of WT and TPKO mice

Figure S5. Generation of adrenal cortex-specific MKK6EE overexpression mice

Figure S6. TP regulated p38 through the Gq/CAMKIIγ signaling in adrenal cortical cells

Figure S7. Mass spectrum results show that 14-3-3γ was phosphorylated at S46 and T70

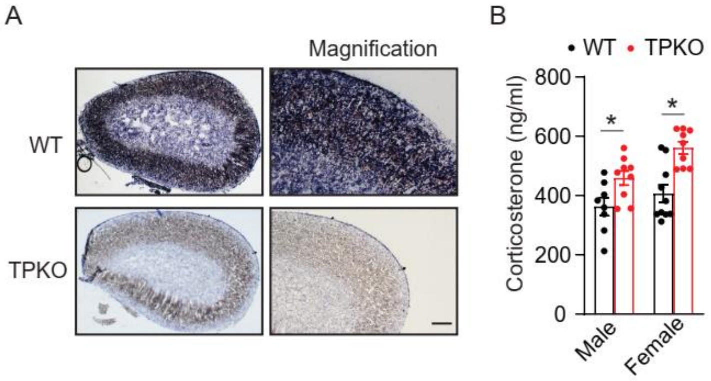

Figure S1. TPKO mice displayed increased corticosterone production. A) *In situ* hybridization showing relative mRNA levels of TP in adrenal glands from mice. Scale bars, 50 µm. B) Corticosterone levels in the plasma of WT and TPKO mice after ACTH administration at 6 PM (*n* = 9–11). For all panels, statistical significance was assessed by unpaired Student's *t-test*. Data are presented as mean ± SEM. *$p < 0.05$ vs. WT.

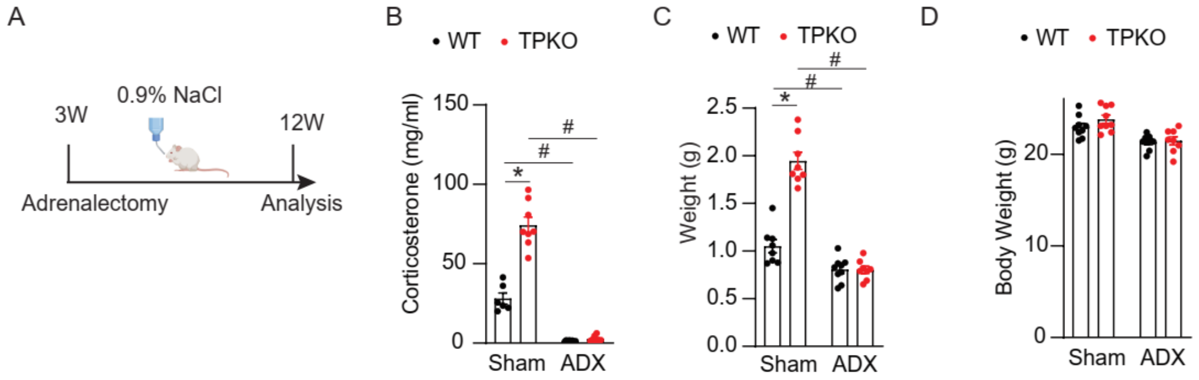

Figure S2. Adrenalectomy decreased corticosterone levels in WT and TPKO mice. A) Schematics for introducing mouse adrenalectomy surgery. B–D) Serum corticosterone levels (B) (n = 6–8), fat mass (C) (n = 8–9), and body weight (D) (n = 8–9) of adrenalectomized (ADX) WT and TPKO mice (n = 8). Statistical significance was assessed by *two-way* ANOVA (B and C). Data are presented as mean ± SEM and $*p < 0.05$ vs WT; $\#p < 0.05$ vs Sham.

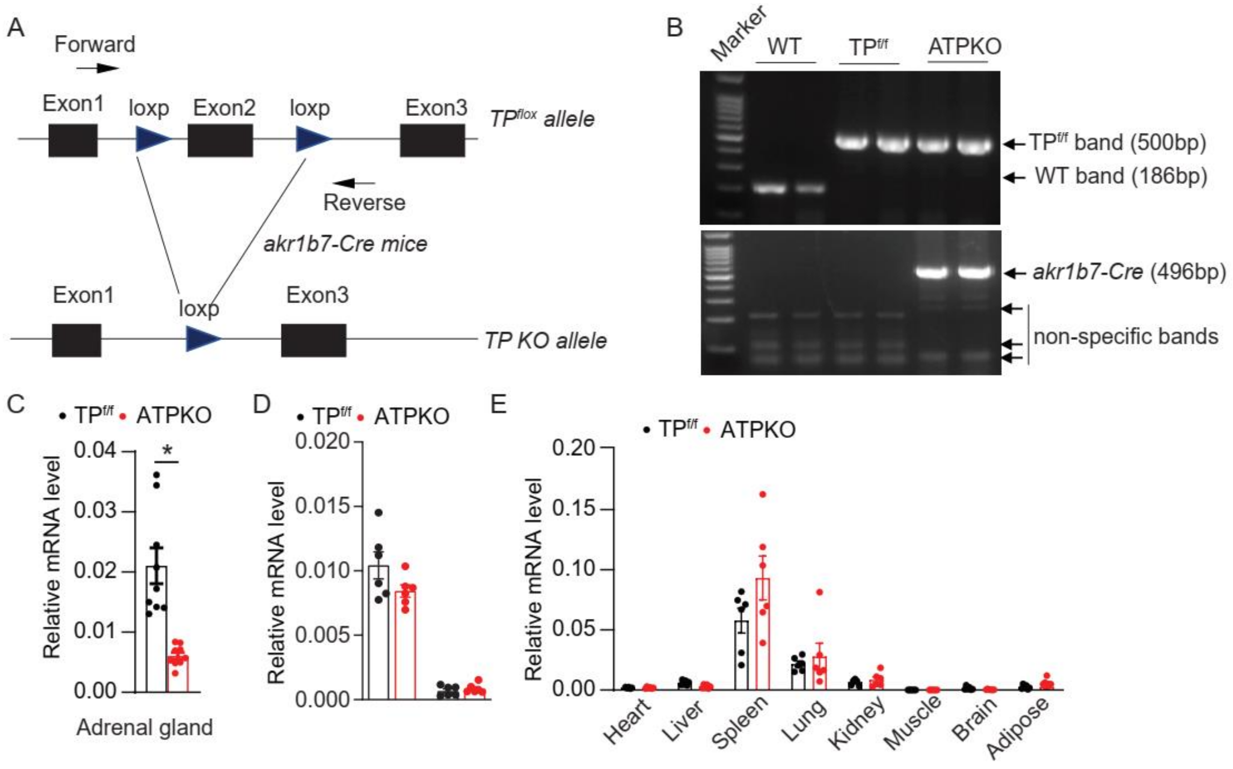

Figure S3. Generation of adrenal cortex-specific TP knockout mice. A) Structure of TP-floxed allele. B) Genotyping of WT, $TP^{flox/flox}$ ($TP^{f/f}$) and adrenal-cortex-specific TP knockout ($TP^{f/f}$ akr1b7-Cre, ATPKO) mice. C–E) mRNA levels of TP in adrenal glands (C) (n = 9), gonadal glands (D) (n = 6) and other tissues (E) (n = 6) of WT and ATPKO mice. Statistical significance was assessed by unpaired Student's *t-test* (C). Data are presented as mean ± SEM and $*p < 0.05$ vs WT.

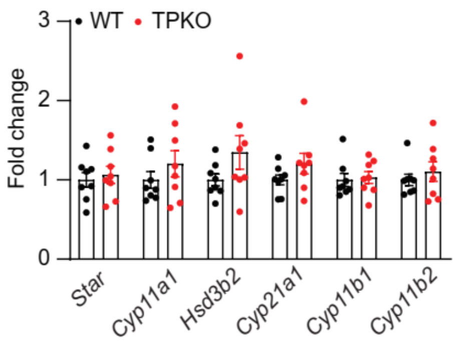

Figure S4. Expression of steroidogenic genes in adrenal glands of WT and TPKO mice. mRNA levels of steroidogenic genes in adrenal glands of male WT and TPKO mice (*n* = 8).

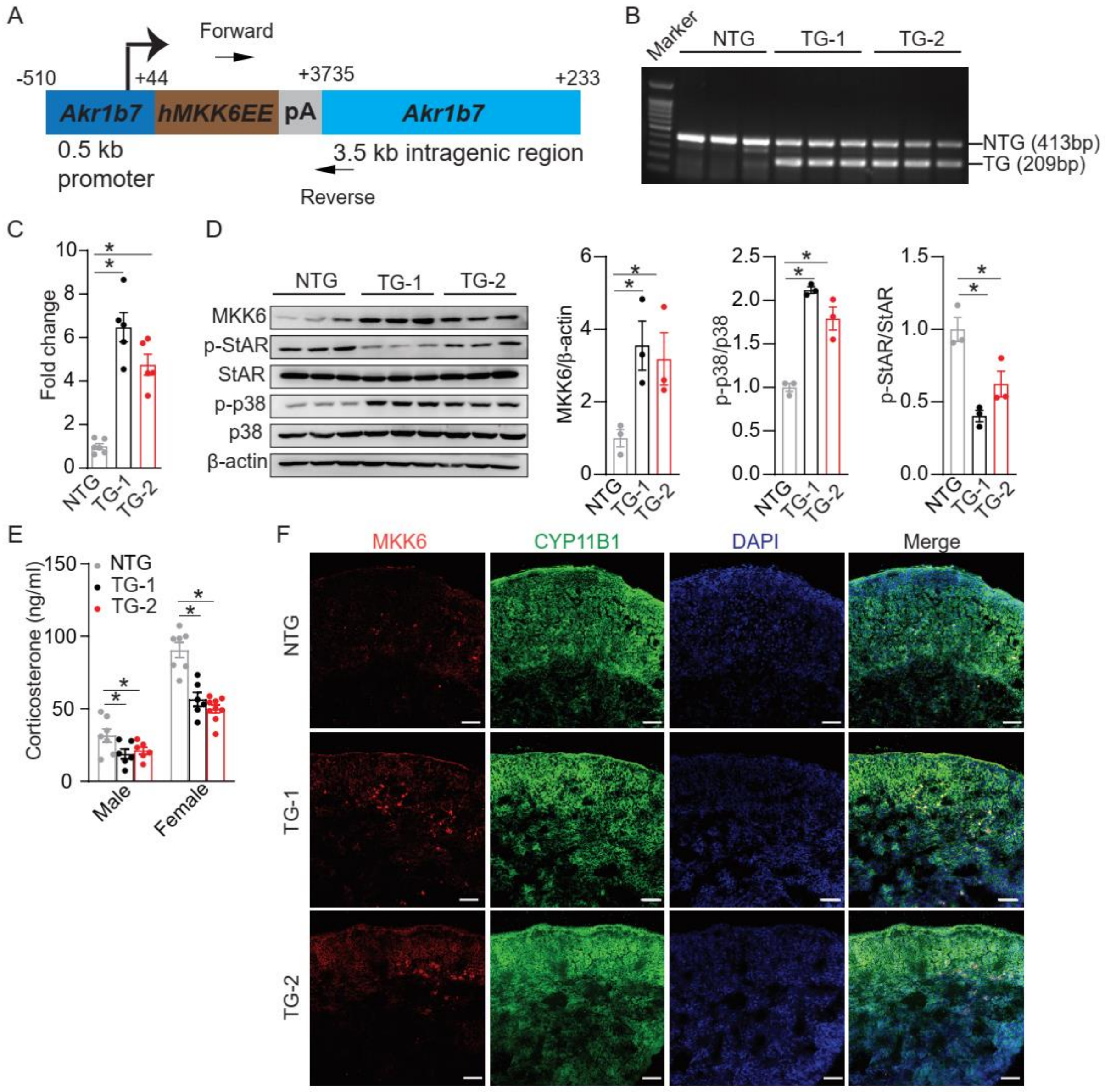

Figure S5. Generation of adrenal cortex-specific MKK6EE overexpression mice. A) Schematic diagram of the construct used for the generation of adrenal cortex-specific mouse MKK6EE overexpression mice. B) Genotyping of non-transgenic mice (NTG) and MKK6(TG) mice. C, D) mRNA levels of MKK6 (C) (n = 5–6) and western blot of indicated proteins (D) in adrenal glands of NTG and TG mice. E) Serum corticosterone levels of NTG and TG mice (n = 6–9). F) Immunofluorescence analysis of CYP11B1 and MKK6 in adrenal glands of NTG and TG mice.

Scale bars, 50 µm. Statistical significance was assessed by unpaired Student's *t-test* (C–E). Data are presented as mean ± SEM and $*p < 0.05$ vs WT.

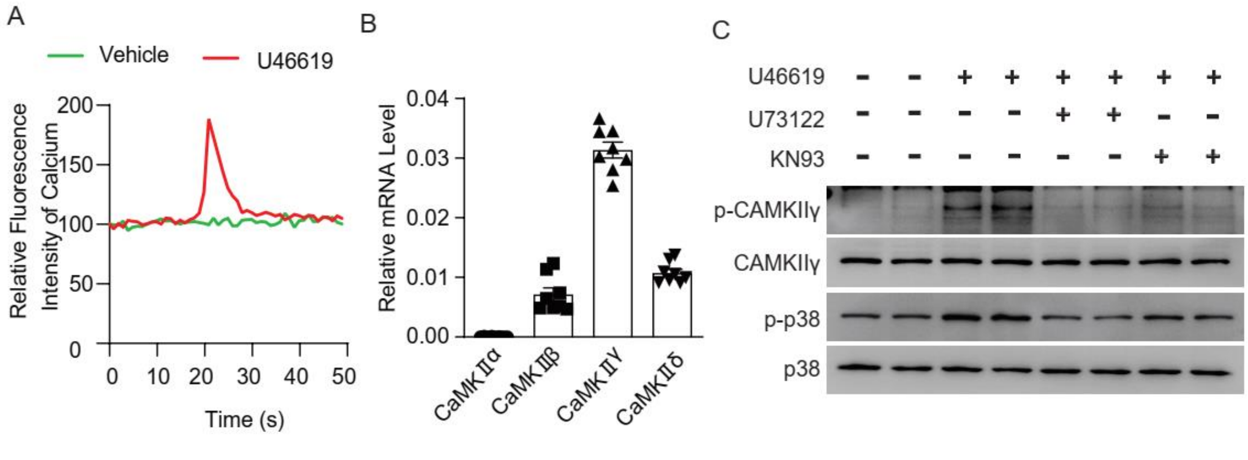

Figure S6. TP regulated p38 through the Gq/CAMKIIγ signaling in adrenal cortical cells. A) Representative calcium fluxes of Y1 cells in response to U46619 (TP agonist, 5 μM) treatment. B) The relative mRNA expression of CaMKII isoforms (α, β, γ, and δ) in adrenal glands (n=8). C) Western blot analysis of p-CAMKIIγ, CAMKIIγ, p-p38 and p38 in U46619(5 μM)-treated Y1 cells in presence of U73122 (PLC inhibitor, 10 μM) and KN93 (CaMKII inhibitor, 10 μM). Data are presented as mean ± SEM.

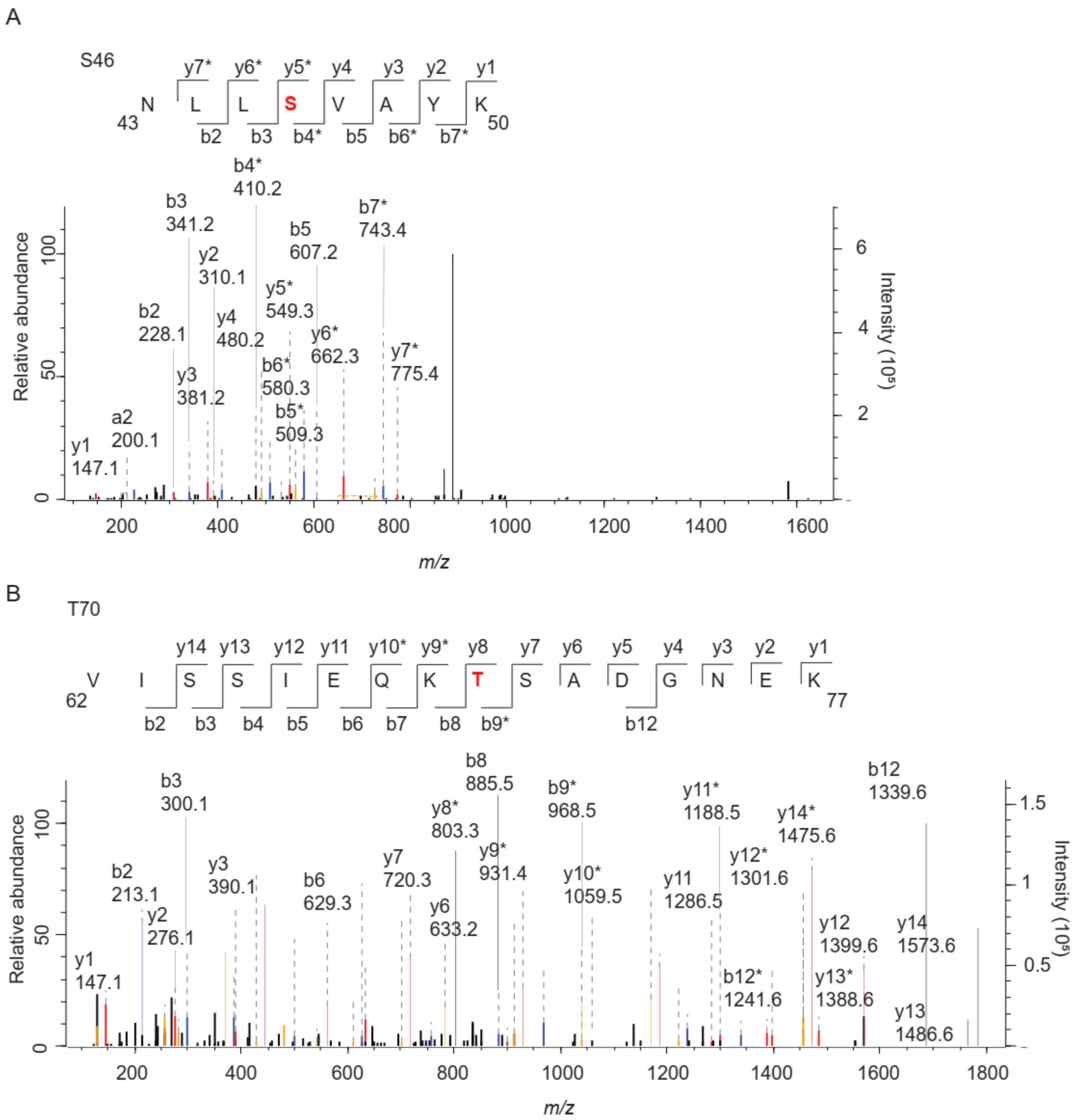

Figure S7. Mass spectrum results show that 14-3-3γ was phosphorylated at S46 (A) and T70 (B).
